# Clustering of health-related behaviours and its relationship with individual and contextual factors in Portuguese adolescents: results from a cross-sectional study

**DOI:** 10.1186/s12887-020-02057-1

**Published:** 2020-05-25

**Authors:** Constança Soares dos Santos, João Picoito, Isabel Loureiro, Carla Nunes

**Affiliations:** 1 Escola Nacional de Saúde Pública, Universidade NOVA de Lisboa, Avenida Padre Cruz, 1600-560 Lisbon, Portugal; 2 Department of Pediatrics, Centro Hospitalar Cova da Beira, Quinta do Alvito, 6200-251 Covilhã, Portugal; 3 Department of Child and Adolescent Psychiatry, Centro Hospitalar e Universitário de Coimbra, Rua Doutor Afonso Romão, 3000-609 Coimbra, Portugal; 4 Centro de Investigação em Saúde Publica, Escola Nacional de Saúde Pública, Universidade NOVA de Lisboa, Avenida Padre Cruz, 1600-560 Lisbon, Portugal

**Keywords:** Health-related behaviours, Adolescents, Cluster patterns, Social determinants, Public health, HBSC

## Abstract

**Background:**

Health behaviours are shaped early in life and tend to occur in complex specific patterns. We aimed to characterise these patterns among Portuguese adolescents and their association with individual and contextual factors.

**Methods:**

This study was based in the Portuguese 2009/10 survey of Health Behaviour in School-Aged Children Study, comprising 4036 adolescents. Individuals were grouped using two-step cluster analysis based on 12 behaviours regarding diet, physical activity, screen use and substance use. The association between clusters and individual and contextual factors was analysed using multinomial regression.

**Results:**

The median age was 13,6, and 54% were female. Overweight and obesity were highly prevalent (25%). We identified four behavioural clusters: “Active screen users”, “Substance users”, “Healthy” and “Inactive low fruit and vegetable eaters”. Sociodemographics varied across clusters. The “Substance users” and “Active screen users” clusters were associated with poor family communication, academic performance and school attachment and violent behaviours, and the “Inactive low fruit and vegetable eaters” were associated with lower socioeconomic status.

**Conclusion:**

The understanding of these health-compromising patterns and their social determinants is of use to Public Health, allowing tailored health-promoting interventions. Further research is needed to understand how cluster membership evolves and its influence on nutritional status.

## Key findings

- We identified four behavioural clusters patterns: “Healthy”, “Substance users”, “Active screen users” and “Inactive low fruit and vegetable eaters”.

- The “Substance users” cluster showed the least favourable social background, with a positive association with poor family communication, academic achievement and school attachment and violent behaviour; followed by “Active screen users” cluster, with a positive association with male gender, bullying and school attachment.

- Each unhealthy pattern suggests different targets for interventions that should take into consideration these social determinants of health.

## Background

Health behaviours are shaped early in life, during childhood and adolescence [1]. Healthy behaviours learned during this critical period lay the foundations of future health [2]. Hence, children and adolescents’ health is regarded as a nation’s wealth [3].

On the other hand, unhealthy behaviours like smoking, alcohol consumption, physical inactivity and unhealthy diet tend to persist into adulthood, contributing to higher risks of non-communicable diseases, like obesity, metabolic syndrome, diabetes and cardiovascular disease [4]. Therefore, they are associated with increased morbimortality and are significant threats to Public Health.

In adolescence, these unhealthy behaviours tend to cluster, with multiple synergic risk factors occurring together [5]. Thus, focusing on these complex clusters rather than on single behaviours may be more effective when planning public health interventions.

Furthermore, these clusters are subject to cultural variation [6]. As a matter of fact, human development and health behaviours are strongly affected by different types of social factors, at the individual, family, community, and national levels [7]. Therefore, the understanding of these behavioural clusters and its relationship with individual and contextual factors is of extreme use to Public Health, allowing tailored health-promoting interventions [8].

There are several studies focusing on the triad eating habits, physical activity and screen-based activities [9] and other studies address substance use [10, 11], but few studies to date take into consideration those four major health determinants together.

In our study, we aimed to identify and characterise patterns of health-related behaviours among Portuguese adolescents and correlate them with individual and contextual factors.

## Methods

### Participants

Data were drawn from the Portuguese 2009/10 survey of Health Behaviour in School-Aged Children (HBSC) study, a WHO cross-sectional study designed to provide information on health behaviours and lifestyles of adolescents aged 11 to 15 years, across different social contexts. Data were collected between Fall 2009 and Spring 2010, using a standardised self-report questionnaire administered in classrooms, following international standards. This national sample is representative of Portuguese adolescents in terms of age, gender and geographic area. The methods used to gather these data are further described in detail elsewhere [12]. The study protocol was approved by the Health Ethics Committee of Hospital de São João, the National Committee on Data Protection and the Ministry of Education, and it meets the ethical requirements of the Helsinki Declaration. Parental approval of children’s participation was mandatory, and all data were gathered anonymously. The overall sample consisted of 4036 adolescents.

### Measures

*Health Behaviours* included 12 physical activity, eating and substance use items, assessed by a self-report questionnaire presented in Table 1.

**Table 1:** Health-behavioural measures included in the analysis

**Health behaviour**	**Response Options**	**Recoded** [13]
**Dietary behaviours**		
“How many times a week do you usually eat or drink …”	7 categories “never”; “< once a week”; “once a week”; “2–4 days a week”; “5–6 days a week”; “once a day”; “every day, more than once	3 categories <= once a week 2–6 days a week daily
Fruits		
Vegetables		
Sweets		
Coke or other soft drinks		
**Physical activity**		
“Over the past 7 days, on how many days were you physically active for a total of at least 60 min per day?”	8 categories 0–7	3 categories 0–2; 3–4; 5–7
**Screen-based activities**		
“About how many hours a day do you usually …”	9 categories “None at all”; “About 1/2 h”; “About 1 h”; “About 2 h”; “About 3 h”; “About 4 h”; “About 5 h”; “About 6”; “About 7 or more.”	3 categories <= 2 h 3–4 h > = 5 h
Watch TV		
Play games		
Use a computer		
**Substance use**		
“Over the last 30 days, on how many occasions have you …”	7 categories “never”, “once or twice”, “3–5 times”, “6–9 times”, “10–19 times”, “20–39 times”, “40 times”.	3 categories Never Once or twice More than twice
Smoked cigarettes		
Drunk alcohol		
Been drunk		
Taken marijuana		

*Physical activity and Sedentary Behaviour* Adolescents who exercised at least an hour a day for five days a week or more were considered physically active, those who exercised three to four days a week were considered inactive and those who exercised two days a week or less were considered highly inactive. Sedentary behaviour included 3 items regarding time spent watching TV, using the computer and playing videogames. Adolescents who spent more than 2 h on those activities were considered sedentary.

*Individual Factors* comprised age, gender and nutritional status, assessed by Body Mass Index (BMI).

Self-reported weight and height were used to calculate BMI (kg/m2). Obesity was defined as BMI greater than the 97th percentile for age and gender, and overweight as BMI between the 85 and 97th percentile, using World Health Organization reference growth charts (Anthro Plus software). Subjects were further classified in two categories “normal weight” / “overweight and obesity”.

*Contextual factors* comprised family, school and peer factors and are presented in Table 2.

**Table 2:** Individual and contextual factors

**Individual factors**	**Response Options**	**Recoded**
Age	Continuous	
Height (self-report)	Body Mass Index	2 categories Normal weight Overweight and obesity
Weight (self-report)		
**Contextual factors**		
**Family factors**		
**Family Affluence Scale**		Sum = 0–9
No. of cars	“No” (0); “One” (1); “Two or more” (2)	dichotomised High (3rd quantile) / Medium-low (1st and 2nd quantiles) Ref: [10, 14]
Own bedroom	“No”(0), “Yes” (1)	
Holiday with family	“Not at all” (0), “Once” (1), “Twice” (2), “More than twice” (3)	
No. of computers at home	“None” (0), “One” (1), “Two” (2), “More than two”(3)	
**Family structure**		
“Check all the people who live in the home where you live all or most of the time.”	“mother”, “father”, “stepmother”, “stepfather”, “grandmother”, “grandfather”, “I live in a foster home”, “other.”	dichotomised Living with both parents / Other family typology Ref: [10]
**Family communication**		
“How easy it is to talk to the following persons about things that really bother you”.	“very easy”, “easy”, “difficult”, “very difficult”, “don’t have or see.”	dichotomised Good communication with both parents (or only parent) / Other Ref: [10]
Mother		
father		
**School factors**		
**School attachment**		
“How do you feel about school at present.”	I like it a lot”, “I like it a bit”, “I don’t like it very much”, “I don’t like it at all”,	dichotomised Like / Dislike Ref: [10]
**Academic achievement**		
“What does your class teacher(s) think about your school performance compared to your classmates”.	“very good”, “good”, “average”, “below average”,	dichotomised Good / Average or below
**Peers factors**		
**No. of evenings a week spent out with friends**	0–7	
**Violent behaviour and victimisation**		
How often / many times have you		dichotomised Yes / No
Taken part in bullying others in the last 2 months	“I haven’t”, “Once or twice”, “2 or 3 times a month”, “once a week”, “several times a week.”	
Being bullied at school in the last 2 months		
Participated in a physical fight in the past 12 months	“I haven’t”, “One time”, “Two times”, “Three times”, “Four times or more.”	

### Statistical analysis

Statistical analysis was done using IBM Statistical Package for the Social Sciences, version 24.0 (SPSS Inc., Chicago, IL). Statistical significance was set to *p* < 0,05.

#### Cluster analysis

Cluster analysis is an exploratory, data-driven method that identifies groups of individuals with similar behaviours, based on the actual structure of the data [15]. In our study, individuals were partitioned into clusters using two-step cluster analysis based on 12 health behaviour variables. Dissimilarity was measured by log-likelihood, with a predetermined maximum number of clusters of 10. The best cluster solution was chosen based on the lowest value of the Schwarz’s Bayesian Criterion (BIC) with significantly high values of BIC change and of ratio of distance measures. Each cluster was further characterised in terms of dimension, age and gender distributions [15].

#### Multinomial regression

The magnitude of the association between individual and contextual factors and cluster membership was further calculated based on crude and adjusted odds ratio (OR) using a multinomial regression (main effect; backward stepwise method; entry and removal test: likelihood ratio; entry probability 0,05; removal probability 0,1) [15].

## Results

### Characteristics of study subjects

The individual and contextual characteristics of the overall sample are presented in Table 3. 53,5% were of the female gender. The median age was 13,58 (Interquartile range 3,50). One-fourth of the overall sample had overweight or obesity (25,1%). The majority lived with both parents (77,7%), 41% had high affluent families, and 59% had medium-low affluent families.

**Table 3:** Individual and contextual characteristics of the overall sample (*n* = 4036)

	n	n (%)	Missing (%)
**Age**^a^	4036	13,58 (3,50); 10,50-16,42^a^	0
**Gender**	4036		0
Male		1878 (46,5%)	
Female		2158 (53,5%)	
**Body Mass Index**	3777		6,4
Normal weight		2830 (74,9%)	
Overweight		729 (19,3%)	
Obesity		218 (5,8%)	
**Family Affluence Scale**	3885		3,7
High		1591 (41,0%)	
Medium		735 (18,9%)	
Low		1559 (40,1%)	
**Family Structure**	4036		0
Living with both parents		3135 (77,7%)	
Other family typology		901 (22,3%)	
**Family Communication**	3786		6,2
Good communication		2142 (56,6%)	
Mixed communication		969 (25,6%)	
Poor communication		675 (17,8%)	
Don’t have or see		35 (0,9%)	
**School Attachment**	4019		0,4
Like		3130 (77,9%)	
Dislike		889 (22,1%)	
**Academic Achievement**	4008		0,7
Good		1981 (49,4%)	
Average		1831 (45,7%)	
Below average		196 (4,9%)	
**Evenings with friends (n° per week)**^a^	3938	0 (1); 0–7^a^	2,4
**Been Bullied last 2 months**	3991		1,1
Never		2498 (62,6%)	
Once or twice		945 (23,4%)	
More than twice		548 (13,6%)	
**Bullied others last 2 months**	3987		1,2
Never		2719 (68,2%)	
Once or twice		898 (22,5%)	
More than twice		370 (9,3%)	
**Participation in a fight last 12 months**	3956		2
Never		2876 (72,7%)	
Once or twice		768 (19,4%)	
More than twice		312,9%)	

Data are presented as n (%) for categorical variables and as Median (Interquartile range); min-max for quantitative variables

^a^ Quantitative variables

### Health behaviours

Prevalence of health behaviours in the overall sample is presented in Table 4.

**Table 4:** Distribution of health behaviours among Portuguese adolescents (*n* = 4036)

Behavioural item				
**Dietary behaviours**	**n**	**once a week or less**	**2 to 6 days/week**	**Daily**
Eat fruits, times/week	**4013**	641 (15,97)	1627 (40,54)	1745 (43,48)
Eat vegetables, times/week	**3998**	975 (24,39)	1921 (48,05)	1102 (27,56)
Eat sweets, times/week	**3999**	1575 (39,38)	1743 (43,59)	681 (17,03)
Drink soft drinks, times/week	**4000**	1687 (42,18)	1440 (36,00)	873 (21,83)
**Physical activity**		**2 days or less**	**3 to 4 days**	**5 days or more**
60 min of physical activity last week, days	**3998**	1505 (37,64)	1287 (20,89)	1206 (30,17)
**Screen-based activities**		**2 h or less**	**3 to 4 h**	**5 h or more**
Watching TV, hours/day	**3792**	1340 (35,30)	2012 (53,10)	440 (11,60)
Videogaming, hours/day	**3815**	2608 (68,40)	988 (25,90)	219 (5,70)
Computer use, hours/day	**3809**	2208 (58,00)	1300 (34,10)	301 (7,90)
**Substance use**		**never**	**once or twice**	**more than twice**
Smoked cigarettes last 30 days, times	**3995**	3552 (88,91)	219 (5,48)	254 (6,36)
Drunk alcohol last 30 days, times	**3976**	2696 (67,81)	865 (21,76)	415 (10,44)
Been drunk last 30 days, times	**3971**	3690 (92,92)	223 (5,62)	58 (1,46)
Cannabis use last 30 days, times	**3926**	3833 (97,63)	41 (1,04)	52 (1,32)

Data are presented in n (%)

43,48% of adolescents ate fruits daily, but only approximately one quarter ate vegetables daily (27,56%), while 60,62% ate sweets, and 57,83% drank soft drinks at least twice a week. Less than one-third of adolescents exercised 5 days per week (30,17%), and only 13,11% (524) reported 60 min of physical activity per day every day. Regarding screen-based activities, 64,70% spent > 2 h per day watching TV, 31,60% spent > 2 h per day playing videogames and 42,00% spent > 2 h using the computer. Regarding substance use, 11,84% had smoked cigarettes, 32,20% had drunk alcohol, 7,08% had been drunk, and 2,36% had used cannabis at least once during last month.

### Cluster groups

Four distinct clusters based on health behaviours were identified. Based on the lowest value of BIC combined with significantly high values of the ratio of BIC change (0,429) and the ratio of distance measures (1713), an interpretable 4 cluster solution was chosen.

#### Cluster characterisation

**Fig. 1:**
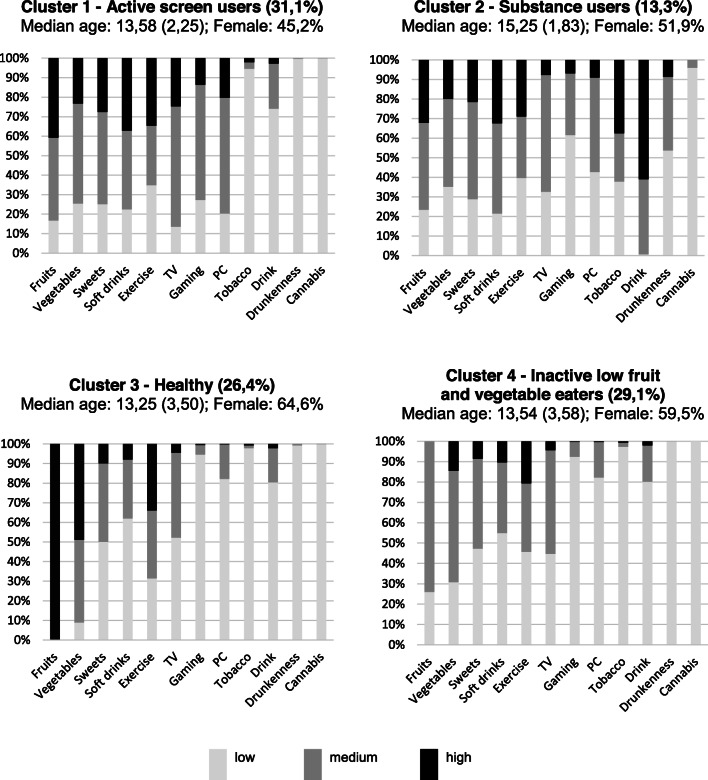
Cluster characterisation. Stacked bar plots showing the distribution of health behaviours in each cluster

As reported in Fig. 1, Cluster 1 had the highest prevalence of screen-based activities and one of the highest prevalence of physical activity, with high consumption of sweets and soft drinks, hence it was named “Active screen users”. Cluster 2 had the highest prevalence of alcohol, tobacco and cannabis use, and was therefore named “Substance users”. Cluster 3 was judged to be the healthiest. It had the highest prevalence of fruits and vegetable consumption and the lowest prevalence of sweet and soft drinks consumption, one of the highest prevalence of physical activity, and low prevalence of screen and substance use, and was therefore named “Healthy”. Cluster 4 had the lowest prevalence of physical activity, with moderate-to-low consumption of fruits and vegetables, low consumption of sweets and soft drinks, hence it was named “Inactive low fruit and vegetable eaters”.

Regarding cluster dimensions, “Active screen users”, “Inactive low fruit and vegetable eaters” and “Healthy” were approximately 30% each, and “Substance users” was the smallest cluster, comprising 13% of adolescents. “Active screen users” cluster was predominantly male (54,8%), “Substance users” cluster comprised older adolescents (median age 15,25), and “Healthy” cluster was predominantly female (64,6%) and younger adolescents (median age 13,25). The between-cluster differences in both median age and gender distributions were statistically significant (*p* < 0,001).

#### Association between individual and contextual factors and cluster membership

**Fig. 2:**
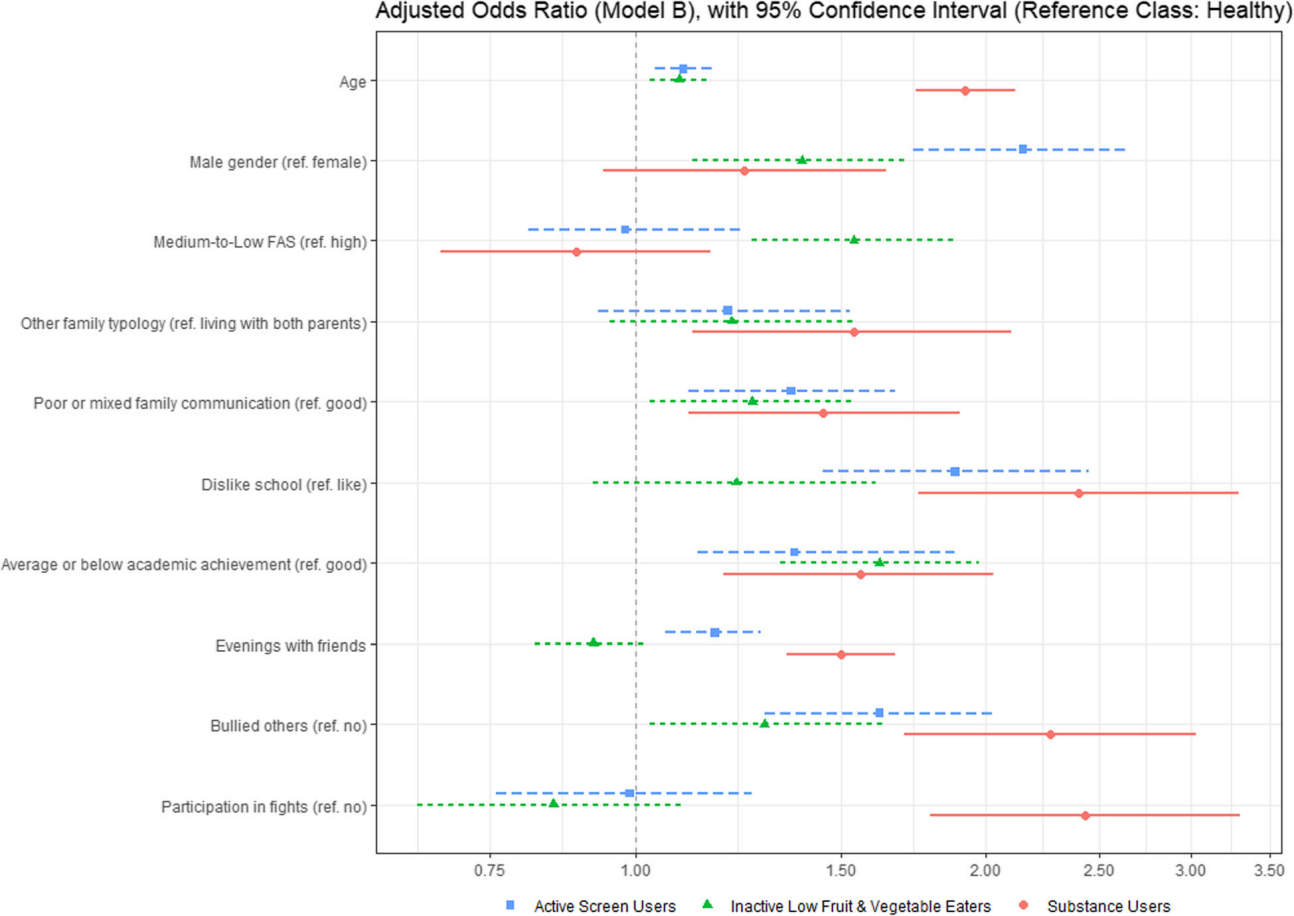
Graphical representation of Adjusted Odds Ratio (Model B), with 95% Confidence Interval. Adjusted for Individual (age, gender) and Contextual factors (family- FAS, family structure and family communication, school - school attachment, academic achievement, and peer - evenings with friends, bullied others, participation in fights)

The association between individual and contextual factors and cluster membership is presented in Table 5. The adjusted odds ratio (model B) is also presented in Fig. 2.

**Table 5:** Crude and adjusted OR between Individual and Contextual factors and Cluster Membership (*n* = 3166)

Variables (reference class)		Cluster Membership (reference cluster: Healthy)					
	OR (CI 95%)	Active Screen users		Substance users		Inactive low fruit and vegetable eaters	
**Age**	Crude OR	**1,16 (1,10-1,22)**	***p*< 0,001**	**1,87 (1,72-2,03)**	***p*< 0,001**	**1,12 (1,07-1,19)**	***p*< 0,001**
	a1	**1,17 (1,11-1,24)**	***p*** **= 0,002**	**1,89 (1,74-2,05)**	***p*< 0,001**	**1,13 (1,07-1,19)**	***p*< 0,001**
	b	**1,10 (1,04-1,17)**	***p*** **< 0,001**	**1,92 (1,74-2,12)**	***p*< 0,001**	**1,09 (1,03-1,16)**	***p*= 0,006**
**Male gender (female)**	Crude OR	**2,22 (1,09-1,69)**	***p*= 0,003**	**1,69 (1,50-2,12)**	***p*< 0,001**	**1,24 (0,67-1,97)**	***p*= 0,002**
	a2	**2,27 (1,89-2,72)**	***p*= 0,004**	**1,86 (1,47-2,35)**	***p*< 0,001**	**1,26 (1,05-1,52)**	***p *= 0,013**
	b	**2,15 (1,73-2,65)**	***p*< 0,001**	1,24 (0,94-1,64)	*p* = 0,131	**1,39 (1,12-1,72)**	***p*= 0,002**
**Overweight/ obesity** **(normal weight)**	Crude OR	1,16 (0,94-1,43)	*p* = 0,171	0,94 (0,72-1,23)	*p* = 0,662	1,17 (0,94-1,44)	*p* = 0,152
**Medium-to-low FAS (High)**	Crude OR	1,06 (0,87-1,26)	*p* = 0,534	0,97 (0,77-1,21)	*p* = 0,761	**1,70 (1,40-2,04)**	***p*< 0,001**
	a	1,09 (0,91-1,30)	*p* = 0,372	0,97 (0,77-1,23)	*p* = 0,802	**1,71 (1,42-2,06)**	***p*< 0,001**
	b	0,98 (0,81-1,23)	*p* = 0,841	0,89 (0,68-1,16)	*p* = 0,382	**1,54 (1,26-1,88)**	***p*< 0,001**
**Other family typology (living with both parents)**	Crude OR	**1,27 (1,02-1,59)**	***p*= 0,032**	**1,80 (1,38-2,33)**	***p*< 0,001**	**1,29 (1,03-1,61)**	***p*= 0,027**
	a	**1,27 (1,01-1,58)**	***p*= 0,041**	**1,81 (1,38-2,39)**	***p*< 0,001**	**1,29 (1,03-1,62)**	***p*= 0,027**
	b	1,20 (0,93-1,53)	p = 0,149	**1,54 (1,12-2,10)**	***p*= 0,007**	1,21 (0,95-1,55)	p = 0,127
**Poor or mixed family communication (good)**	Crude OR	**1,33 (1,10-1,59)**	***p*= 0,003**	**2,00 (1,56-2,52)**	***p*< 0,001**	**1,40 (1,16-1,68)**	***p*< 0,001**
	a	**1,48 (1,22-1,79)**	***p*< 0,001**	**1,78 (1,39-2,27)**	***p*< 0,001**	**1,40 (1,16-1,70)**	***p*= 0,001**
	b	**1,36 (1,11-1,67)**	***p*= 0,003**	**1,45 (1,11-1,90)**	***p*= 0,007**	**1,26 (1,03-1,54)**	***p*= 0,028**
**Dislike school (like)**	Crude OR	**2,56 (2,01-3,25)**	***p*< 0,001**	**4,25 (3,24-5,59)**	***p*< 0,001**	**1,60 (1,25-2,07)**	***p*< 0,001**
	a	**2,30 (1,81-2,94)**	***p*< 0,001**	**3,70 (2,79-4,91)**	***p*< 0,001**	**1,53 (1,18-1,97)**	***p*= 0,001**
	b	**1,88 (1,45-2,45)**	***p*< 0,001**	**2,40(1,75-3,29)**	***p*< 0,001**	1,22 (0,92-1,61)	p = 0,172
**Average or below Academic Achievement (good)**	Crude OR	**1,65 (1,38-1,97)**	***p*< 0,001**	**2,36 (1,88-2,96)**	***p*< 0,001**	**1,85 (1,55-2,21)**	***p*< 0,001**
	a	**1,57 (1,31-1,88)**	***p*< 0,001**	**1,94 (1,53-2,46)**	***p*< 0,001**	**1,78 (1,48-2,14)**	***p*< 0,001**
	b	**1,37 (1,13-1,88)**	***p*= 0,001**	**1,56 (1,19-2,03)**	***p*= 0,001**	**1,62 (1,33-1,97)**	***p*< 0,001**
**Evenings with friends**	Crude OR	**1,39 (1,22-1,46)**	***p*< 0,001**	**1,73 (1,58-1,90)**	***p*< 0,001**	0,97 (0,88-1,08)	*p* = 0,581
	a	**1,24 (1,13-1,34)**	***p*< 0,001**	**1,63 (1,48-1,79)**	***p*< 0,001**	0,93 (0,84-1,03)	*p* = 0,165
	b	**1,17 (1,06-1,28)**	***p*< 0,001**	**1,50 (1,35-1,67)**	***p*< 0,001**	0,92 (0,82-1,02)	*p* = 0,118
**Been bullied (no)**	Crude OR	**1,44 (1,20-1,73)**	***p*< 0,001**	**1,42 (1,13-1,80)**	***p*= 0,003**	1,18 (0,98-1,42)	*p* = 0,084
	a	**1,36 (1,13-1,64)**	***p*< 0,001**	**1,58 (1,25-2,03)**	***p*< 0,001**	1,18 (0,98-1,43)	*p* = 0,083
	b	**–**		**–**		–	
**Bullied others (no)**	Crude OR	**1,95 (1,60-2,38)**	***p*< 0,001**	**2,88 (2,27-3,65)**	***p*< 0,001**	**1,29 (1,05-1,59)**	***p*= 0,015**
	a	**1,81 (1,48-2,22)**	***p*< 0,001**	**3,35 (2,59-4,32)**	***p*< 0,001**	**1,29 (1,05-1,59)**	***p*= 0,017**
	b	**1,62 (1,29-2,03)**	***p*< 0,001**	**2,27 (1,70-3,03)**	***p*< 0,001**	1,29 (1,03-1,64)	*p* = 0,058
**Participation in fights (no)**	Crude OR	**1,61 (1,30-1,98)**	***p*< 0,001**	**3,16 (2,48-4,04)**	***p*< 0,001**	0,98 (0,78-1,23)	*p* = 0,856
	a	**1,34 (1,08-1,68)**	***p*< 0,001**	**3,84 (2,93-5,04)**	***p*< 0,001**	0,98 (0,94-1,19)	*p* = 0,592
	b	0,99 (0,76-1,26)	*p* = 0,906	**2,43 (1,79-3,30)**	***p*< 0,001**	0,85 (0,65-1,10)	*p* = 0,218

(a) adjusted for individual factors (age and sex); (a1) adjusted for gender, (a2) adjusted for age

(b) adjusted for individual + contextual factors (FAS, family structure and family communication, school attachment, academic achievement, evenings with friends, bullied others, participation in fights)

*FAS* Family Affluence Scale

Older adolescents were more likely to be “Substance users”, and male adolescents were twice more likely to be “Active screen users”, comparing to “Healthy”.

We found no association between nutritional status and cluster membership.

Socioeconomic status had no relationship with cluster membership except for the “Inactive low fruit and vegetable eaters” cluster. Adolescents from medium-to-low affluent families were more likely to be “Inactive low fruit and vegetable eaters”, even after adjusting to individual and contextual factors.

Adolescents not living with both parents had higher odds of being “Substance users”, even after adjusting to individual and other contextual factors. In “Active screen users” and “Inactive low fruit and vegetable eaters” cluster, this association disappeared after adjusting to other contextual factors.

Adolescents who reported poor family communication had higher odds of being “Substance users”, “Inactive low fruit and vegetable eaters” and “Active screen users”, even after adjusting to individual and contextual factors.

Regarding school factors, adolescents with a poor school attachment were more likely to be “Substance users” and to be “Active screen users”. A poor academic achievement was also associated with higher odds of belonging to “Substance users”, “Inactive low fruit and vegetable eaters” and “Active Screen users” clusters.

Regarding peer factors, the number of evenings spent with friends was positively associated with the “Substance users” and “Active screen users” clusters. Adolescents who had been bullied had a higher risk of belonging to the “Substance users” and “Active screen users” clusters, but these associations disappeared after adjusting to other factors. Adolescents who had bullied others were more likely to be “Substance users” and “Active screen users”, even after adjusting for other factors. Fighting was also positively associated with “Substance users” cluster, even after adjustment. We found no association between peer factors and the “Inactive low fruit and vegetable eaters” cluster, except for bullying others, but this association disappeared after adjusting for other factors.

## Discussion

Our sample showed a high prevalence of overweight and obesity and well as a high prevalence of unhealthy behaviours. A high proportion of adolescents showed low consumption of fruits and vegetables (15,97% of adolescents consume fruits once a week or less, and 24,39% consume vegetables once a week or less) and high consumption of sweets and soft drinks. Moreover, it is alarming that only 13,11% of the overall sample met the international physical activity recommendations of one hour per day [16], 37% being highly inactive. Furthermore, physical inactivity was prevalent across all clusters. In fact, Portuguese adolescents, especially girls, are persistently among the most physically inactive youth in Europe [17, 18]. Regarding substance use, we found a lower prevalence of smoking (12% vs 19%); alcohol drinking (32% vs 42%) and cannabis consumption (2,36% vs 8%) compared to adolescents included in 2015 Portuguese ESPAD study, although the latter comprised older (13 to 18-year-old) adolescents [19].

### Cluster patterns and individual factors

We found 4 clusters, namely “Active screen users”, “Substance users”, “Healthy” and “Inactive low fruit and vegetable eaters”, each with unique behavioural patterns.

A study based on the same HBSC Portuguese dataset focused on a narrower subset of variables regarding diet, physical activity and screen use. It used k-means cluster analysis and found 3 clusters (“active gamers”, “healthy” and “sedentary”) [20].

In our study, we opted to include other risk factors like alcohol, tobacco and cannabis use alongside with diet, exercise and screen use, since these health-compromising behaviours tend to co-occur and may have a synergistic effect on health. Furthermore, we used a two-step cluster analysis, which better handles ordinal variables. In contrast, k-means is limited to continuous data and is based on a predetermined number of clusters.

One recent review focusing on clustering of diet, physical activity and sedentary activities reported that the most common cluster pattern observed was mixed physical activity with sedentary activities (either high levels of both or low levels of both). This study suggests that high levels of physical activity can coexist with high levels of sedentary behaviour, as in the “Active screen users” cluster we found [9].

Most studies show smoking clusters with alcohol abuse in complex ways [10, 21]. One study in Italy using HBSC data found 6 clusters (“smoking drinker”, “non-drinking smoker”, “quasi-healthy”, “symptomatic”, “violent” and “screen passion”) [22]. Similarly, in our study alcohol and tobacco use both clustered in the same group (“Substance users”), comprising older adolescents.

The same review concluded that younger children tended to be in the healthiest clusters regarding both diet and physical activity, as it happens in our “Healthy” cluster [9].

We also found that the “Healthy” cluster was predominantly female and that boys were twice more likely to be “Active screen users” and more likely to be “Substance users”, although the latter association disappeared after adjusting to contextual factors. In fact, gender differences in cluster patterns have been reported in several studies, showing a consistent trend that boys were more likely to be in high screen-time clusters and girls tended to be in lower physical activity/ healthier diet clusters [23].

Surprisingly, we found no association between BMI and cluster membership. This may be due to the fact that BMI was calculated using self-report data. Furthermore; overweight and obese adolescents, especially those being treated, may tend to report healthier eating patterns according to what is socially expected of them, not their current habits [24]. Also, the high prevalence of physical inactivity we found across all clusters may contribute to attenuate BMI differences between clusters.

### Clustering patterns and family factors

In our study, lower socioeconomic status was associated with “Inactive low fruit and vegetable eaters” cluster. Previous research confirms that adolescents from lower affluent families are less likely to engage in moderate to vigorous physical activity, sports and other outdoor extracurricular activities [25]. Also, they tend to live in less walkable neighbourhoods [26]. Furthermore, adolescents from lower socioeconomic backgrounds tend to report lower fruit and vegetable intake and are more likely to attend schools surrounded by calorie-dense and nutrient-poor fast food stores [27, 28]. We found no association with substance use, to which a low socioeconomic status has been traditionally associated [29]. In fact, conflicting evidence has been reported in the literature. A meta-analysis focusing on marijuana and alcohol use and socioeconomic status found higher rates of substance use among lower socioeconomic status [30].

On the other hand, a literature review reported that low socioeconomic status was associated with more inadequate diets, lower levels of physical activity, and higher cigarette smoking, but found no clear association with alcohol and cannabis consumption [31]. Two recent studies found a positive association between socioeconomic status and smoking [32, 33]. These conflicting results may reflect the complex interactions between exposition to risk behaviours in family and peers, access, and having money to spend, factors that we have not accounted for in our study [32, 33].

Regarding family structure, in our study, adolescents not living with both parents had higher odds of belonging to “Substance users” cluster, even after adjusting to other factors. Other family typologies, namely monoparental families, are at higher risk of financial strain, lower socioeconomic status, psychological stress, and thus undesired health outcomes [34]. Nonetheless, in our study, this association remained significant even after adjusting to socioeconomic status.

Also, adolescents who reported mixed or poor family communication had higher odds of belonging to an unhealthy cluster, even after adjusting to other factors. A recent review focusing on parenting factors concluded that family attachment and communication are protective against substance use during adolescence [35]. Previous research addressing the intricate relationship between different family factors also suggests that family structure and family communication are both associated with health behaviours and outcomes, regardless of socioeconomic status [36].

### Clustering patterns and school and peer factors

Regarding school factors, an average or below-average academic achievement was associated with higher odds of belonging to an unhealthy cluster. Several studies support that there is a positive relationship between health and education, and improving students health behaviours, namely diet, physical activity, sleep, screen time, and nutritional status, has shown to improve academic achievement [37, 38].

Also, adolescents with poor school attachment were more likely to be “Substance users” and “Active screen users”. Indeed, high social connectedness is associated with better health and subjective wellbeing, especially for family, followed by school, peers and community [39]. Moreover, school attachment increases engagement with norms and improves health behaviours, reduces the risk of internalising disorders and substance use and, in turn, leads to better health and wellbeing [40, 41]. In our study, violent behaviour (bullying and fighting), but not victimisation, were also positively associated with the “Substance users” and “Active screen users” clusters. Previous research has consistently associated violence with unhealthy behaviours, substance use, sexual risk-taking and deviant behaviour during adolescence and later in life [42].

### Strengths and limitations

This study provided new evidence about the relationship between individual and contextual factors and clustering of health behaviours. To date, this is one of few studies in Portugal that explicitly addressed this relationship and that included substance use besides eating habits, exercise and screen use. Although data collection was based on a self-report questionnaire, its psychometric properties were studied and improved over the years in several different countries. Several studies have shown that self-report measures are highly reliable and accurate when questions are self-administered, in a school setting and anonymous, even for soft issues like substance use [12]. We analysed a broad range of individual and contextual covariates and all variables included in our study showed low proportions of missing data.

However, this study has some limitations. Unfortunately, it did not collect information from other sources (like parental report) nor objective measures of physical activity, sedentary time and substance use were available. On the other hand, it is well known that many unhealthy habits of adolescents correlate with unhealthy habits of their parents, regarding eating behaviour, sedentary behaviour and physical activity, even after adjusting for gender and socioeconomic background [43, 44]. Also, one of the most important predictors of substance use during adolescence is parental substance use [45]. Therefore, it would have been important to collect information about parental health behaviours.

Since it is a data-driven method, cluster analysis has few adjustment indexes, and one might argue that there is little evidence of cluster existence. Also, we recategorized health behaviour variables according to their distributions (due to the low number in extreme categories), according to previous research, and, whenever possible, to international recommendations. Nevertheless, our cluster solution may be biased by this recategorization.

Although it is a large national representative sample in terms of age, gender and geographic area, and collected in a school setting which lowers the risk of selection bias, we must bear in mind that health-related behaviours are subject to cultural variation that may hinder generalisation. Furthermore, it is a cross-sectional study, which does not allow to establish causality nor its direction. In fact, there may be dual-direction effects between health behaviours and contextual factors. For instance, school attachment, substance use and delinquency mutually reinforce each other over time [46]. Also, although poor family attachment and communication are risk factors for substance use during adolescence [35], there is also evidence that adolescent substance use is a predictor of physical and psychological aggression against parents, possibly because of the direct effects (pharmacological, neurotoxic, and withdrawal), conflicts and discussions over money, and shared causes for substance use and aggression [47]. Together, these studies support the reciprocal interaction between health behaviours and the social environment, evidencing that adolescents influence their social environment and in turn, are influenced by it [48].

### Conclusions and implications

Cluster analysis identified three major health-compromising behaviour patterns, with different relations with individual and contextual factors. The identification and characterisation of these specific groups are key steps for comprehensive public health policies. A review focusing on behavioural change during adolescence through school-based interventions concluded that most interventional studies target one of two groups of behaviours: substance use (drugs, alcohol and tobacco use) and energy balance (eating behaviours, physical activity, and screen-based activities) [49]. However, targeting different behavioural domains simultaneously has a synergistic effect, since unhealthy behaviours share a common core of social determinants [50, 51].

Another review focusing on health promotion interventions on adolescents using an ecological framework concluded that they are effective, but their effect is somewhat small, evidencing the need to identify further key aspects of the social environment that influence health behaviours [52].

In our study, poor family communication and poor school attachment and academic performance were associated with “Active screen users” and “Substance users” clusters and violent behaviour was associated with “Substance users” cluster, even after adjusting to socioeconomic status. Hence, our study points out that family communication, academic performance, school attachment and violent behaviours are possible areas for family and school-based health-promoting interventions. Other studies have demonstrated that interventions promoting positive interactions and effective communication between family members and between teachers and students help to develop a sense of belonging to families, schools, and communities and may promote healthier behaviours in adolescence [53–55].

Therefore, these results may serve as a basis to tailored health-promoting interventions, that should address multiple health behaviours, involve adolescents, their families and the community and focus on family communication and school attachment. Further longitudinal research is needed to understand how cluster membership evolves during childhood and adolescence, how these behavioural clusters differ over time and across countries and socio-economic contexts, and its influence on health outcomes, namely nutritional status.

## Data Availability

The data that support the findings of this study are available from HBSC Data Management Centre repository [http://hbsc-nesstar.nsd.no/webview/], but restrictions apply to the availability of these data, which were used under license for the current study. Data are available upon written permission or license obtained from the HBSC Data Bank Manager.

## Abbreviations

BIC: Schwarz’s Bayesian Criterion
BMI: Body Mass Index
FAS: Family Affluence Scale
HBSC: Health Behaviour in School-Aged Children
OR: Odds ratio
WHO: World Health Organization
